# La cytostéatonécrose du nouveau-né: à propos de deux observations

**DOI:** 10.11604/pamj.2015.22.34.6957

**Published:** 2015-09-16

**Authors:** Sahar Messaoudi, Anass Es Seddiki, Mounia Chaalal, Rim Amrani

**Affiliations:** 1 Service de Néonatologie, CHU Mohamed VI, Faculté de Médecine et de Pharmacie d'Oujda, Université Mohammed 1^er^, Oujda, Maroc; 2 Cabinet de Pédiatrie privé, Boulevard Allal Ben Abdellah, Angle Idriss Al Akbar, Oujda, Maroc

**Keywords:** Cytostéatonécrose, Nouveau-né, Hypercalcémie, Néphrocalcinose, fat necrosis, newborn, Hypercalcemia, Nephrocalcinosis

## Abstract

La cytostéatonécrose est une lésion de survenue rare dont la pathogénie est incomplètement connue. Elle se présente sous la forme de placards cutanés indurés et violacés sur peau claire ou hyperchromiques sur peau noire, localisés souvent au niveau de la face, du tronc, des fesses et de la racine des membres. Probablement due à une anomalie des tissus graisseux: trouble du métabolisme des graisses avec excès de graisses saturées dans le tissu sous-cutané, hypoxie par souffrance néonatale ou hypothermie favorisant la cristallisation des graisses saturées et la nécrose graisseuse. L’évolution de la cytostéatonécrose est en règle bénigne. Mais certaines complications (l'hypocalcémie et les troubles métaboliques) peuvent survenir et engendrer le pronostic vital. Nous rapportons deux cas de cytostéatonécrose néonatale néonatale précoce dans le but est de décrire cette symptomatologie et de préciser l’évolution des lésions à moyen terme.

## Introduction

La cytostéatonécrose du nouveau-né (CSN) est une hypodermite aiguë se développant durant les premiers jours de vie [1]. Elle se présente sous la forme de placards cutanés indurés et violacés sur peau claire ou hyperchromiques sur peau noire, localisés souvent au niveau de la face, du tronc, des fesses et de la racine des membres. Sur le plan histologique, on retrouve sous un épiderme et un derme normaux, une panniculite lobulaire avec des foyers de nécrose éosinophile du tissu adipeux englobant des fentes radiaires intra-adipocytaires optiquement vides, correspondant à une dissolution et une cristallisation lipidique [2]. Les principales situations à risque classiquement rapportées sont la macrosomie foetale souvent dans un contexte de mère diabétique, l'asphyxie périnatale, l'hypothermie sévère et les traumatismes tissulaires au cours de manœuvres instrumentales ou au cours de la réanimation néonatale [2–5]. L’évolution de la cytostéatonécrose est en règle bénigne. Cependant, dans certains cas, la survenue d'une hypercalcémie sévère, peut être source de certaines complications engageant parfois le pronostic vital [4–6]. Nous rapportons deux observations de cytostéatonécrose néonatale néonatales avec une description du contexte de survenue et de l’évolution à moyen terme.

## Patient et observation

### Observation 1

Il s'agit d'un nouveau-né de sexe masculin, né à terme, par voie basse dans un contexte d'asphyxie périnatale avec un liquide amniotique purée de pois. Une manoeuvre d'expression abdominale a été réalisée par la sage-femme, pour favoriser l'expulsion. L'enfant a été réanimé. Le poids de naissance a été de 4200 g. Il a été référé pour sclérème cutané constaté par les parents à jours 10 de vie. L'observation des lésions montrait des placards érythémateux faisant évoquer une cytostéatonécrose néonatale de localisation dorsale et scapulo-axillaire droite et aux bras. La calcémie était normale de même que l’échographie abdominale. Il a été mis sous surveillance avec non supplémentation en vitamine D. À 2 mois de vie, les lésions cutanées avaient complètement régressé sans séquelles.

### Observation 2

**Figure 1:**
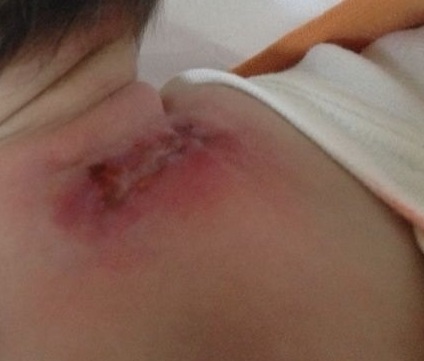
Abcès cutanées en cours de cicatrisation de localisation dorsale évoquant la cytostéatonécrose néonatale

Il s'agit d'un nouveau-né de sexe féminin, né à terme par voie basse dans un contexte de souffrance foetale aigue, avec un poids de naissance de 4700 g, ayant nécessité une réanimation à la naissance, le score d'apgar était à 3/6/8. Les lésions ont été constatées à J12 de vie. Il s'agit de placards inflammatoires indurés cervico-dorsaux, évoquant une cytostéatonécrose (Figure 1). La calcémie était normale. Il y avait un syndrome inflammatoire avec une CRP à 26 mg/L et une leucocytose à 20 500/mm3. Il a bénéficié également d'une simple surveillance avec non supplémentation en vitamine D. Le nouveau-né a été revu à 1 mois de vie, on avait noté une abcédation avec ouverture de la plaie, le contrôle de calcémie était correcte. La lésion a été nettoyée. L’évolution a été favorable au bout d'un mois de traitement symptomatique. A noter que les deux nouveaux nés n'ont pas reçu la dose de charge de la vitamine D, normalement introduite à J 10 de vie à l'occasion du premier vaccin selon le calendrier national de vaccination, ce qui a sûrement prévenu la survenue d'hypercalcémie.

## Discussion

Nous avons décrit ces deux cas de CSN néonatale précoce dans le but de décrire la symptomatologie et de préciser l’évolution des lésions à moyen terme. La CSN néonatale est une lésion de survenue rare dont la pathogénie est incomplètement connue. Plusieurs hypothèses ont été émises en rapport avec une anomalie des tissus graisseux: trouble du métabolisme des graisses avec excès de graisses saturées dans le tissu sous-cutané, hypoxie par souffrance néonatale ou hypothermie favorisant la cristallisation des graisses saturées et la nécrose graisseuse [1, 7]. Cette pathogénie explique les facteurs de risque généralement incriminés et que nous avons observés de façon évidente dans nos deux observations. Il s'agit de l'asphyxie périnatale, la notion de traumatisme obstétrical, de réanimation avec des manoeuvres inadaptées, les compressions abdominales [8, 9]. D'autres facteurs sont été incriminés; l'hypothermie thérapeutique qui est devenue d'indication large dans les asphyxies périnatales sévères est actuellement souvent décrite comme cause de CSN [2]. D'autres facteurs maternels tels que le diabète gestationnel, la pré-éclampsie, le placenta prævia, la prise de cocaïne ou d'inhibiteurs calciques, l'incompatibilité dans le système rhésus sont rapportés [10, 11].

Dans d'autres cas, un terrain lié à l'enfant a été suspecté: il s'agit en particulier des dyslipidémies familiales ou des thrombophilies, notamment le syndrome des antiphospholipides [1]. Bien que la CSN en période néonatale soit très bien décrite, sa survenue reste très rare. Le diagnostic clinique est souvent méconnu et l'aspect des lésions peut faire évoquer d'autres lésions telles qu'un sclérème, une cellulite ou une myosite. Les lésions apparaissent en général précocement durant la première semaine de vie [3, 10, 12]. Elles débutent par un érythème qui laisse rapidement place à des zones d'hypodermite rouge violine plus ou moins diffus, sous forme de placards indurés, violacées ou rouges, souvent douloureuses. La topographie des lésions observées chez nos malades rejoint celle qui est rapportée dans les observations cliniques, en particulier leur localisation au niveau des fesses, des cuisses, du tronc et des joues [4, 5]. La CSN est en règle bénigne avec une évolution vers la guérison sans séquelle. Cliniquement l'inflammation locale s'atténue progressivement, l'infiltrat graisseux régresse plus lentement en quelques semaines à quelques mois pour laisser place à une atrophie du tissu sous-cutanée de durée variable [1, 6, 10]. Chez nos patients, la régression des lésions était obtenue entre 2 et 6 mois. L’évolution vers une abcédation précoce des lésions, comme dans notre deuxième observation, ne semble pas être décrite. En réalité, la seule crainte au cours d'une CSN néonatale est la survenue d'une hypercalcémie sévère. la non prise de la dose de charge de la vitamine D a pu prévenir la survenu de cette hypercalcémie [2, 3, 6, 10, 12]. Cette hypercalcémie peut se manifester par des difficultés de tétées, des vomissements, une anorexie, une agitation [6, 12]. Elle survient volontiers dans les formes disséminées de CSN, ce n’était pas le cas dans nos deux observations [4]. Le mécanisme de survenue de l'hypercalcémie n'est pas clair, plusieurs mécanismes pouvant être intriqués [4, 5]: nécrose des cellules adipeuses entraînant une augmentation des prostaglandines avec activation des ostéoclastes; largage de calcium par les adipocytes nécrosés; production anormale de 1,25-dihydroxyvitamine D par les macrophages augmentant le turnover osseux. Le risque d'hypercalcémie est directement corrélé à l'extension des lésions cutanées. L'hypercalcémie majeure comporte le risque de dépôts tissulaires, en particulier la néphrocalcinose [4, 10]. Des dépôts cardiaques (septum inter auriculaire, valves), hépatiques et dans la veine cave inférieure ont aussi été décrits [1]. Ainsi au cours de l’évolution d'une CSN, le pronostic dépend de l'existence ou non de cette hypercalcémie. Il est donc recommandé de monitorer la calcémie tant que persiste les lésions et même après leur régression. En effet des cas d'hypercalcémie tardive ont été rapportés [5]. Lorsque le taux de calcémie est menaçant (> 120 mg/L) ou que l'enfant est symptomatique, les thérapeutiques préconisées sont l'hyperhydratation associée à un diurétique thiazidique [4, 5], la corticothérapie ou les biphosphonates pour passer la phase aiguë [3]. Cette thérapie associée à l'arrêt des apports de vitamine D a été également jugée efficace dans d'autres observations cliniques rapportées [4, 5]. Pour les cas d'hypercalcémie légère, des apports réduits en vitamine D avec un apport adéquat de matières grasse riche en triglycérides à chaîne moyenne permettent en général une régression spontanée de l'hypercalcémie.

## Conclusion

La cytostéatonécrose néonatale est une affection bien décrite mais rarement rencontrée en pratique courante et donc souvent méconnue par les praticiens. Elle est généralement d’évolution bénigne. La principale complication est l'hypercalcémie qui est souvent asymptomatique mais peut parfois être menaçante, justifiant une surveillance prolongée du taux de calcium, si possible jusqu’à la disparition des lésions cutanées. L’évolution vers la surinfection et l'abcédation des lésions est possible mais semble rare.
